# A Reduce and Replace Strategy for Suppressing Vector-Borne Diseases: Insights from a Stochastic, Spatial Model

**DOI:** 10.1371/journal.pone.0081860

**Published:** 2013-12-20

**Authors:** Kenichi W. Okamoto, Michael A. Robert, Alun L. Lloyd, Fred Gould

**Affiliations:** 1 Department of Entomology, North Carolina State University, Raleigh, North Carolina, United States of America; 2 Department of Mathematics and Biomathematics Graduate Program, North Carolina State University, Raleigh, North Carolina, United States of America; 3 Fogarty International Center, National Institutes of Health, Bethesda, Maryland, United States of America; Centro de Pesquisas René Rachou, Brazil

## Abstract

Two basic strategies have been proposed for using transgenic *Aedes aegypti* mosquitoes to decrease dengue virus transmission: population reduction and population replacement. Here we model releases of a strain of *Ae. aegypti* carrying both a gene causing conditional adult female mortality and a gene blocking virus transmission into a wild population to assess whether such releases could reduce the number of competent vectors. We find this “reduce and replace” strategy can decrease the frequency of competent vectors below 50% two years after releases end. Therefore, this combined approach appears preferable to releasing a strain carrying only a female-killing gene, which is likely to merely result in temporary population suppression. However, the fixation of anti-pathogen genes in the population is unlikely. Genetic drift at small population sizes and the spatially heterogeneous nature of the population recovery after releases end prevent complete replacement of the competent vector population. Furthermore, releasing more individuals can be counter-productive in the face of immigration by wild-type mosquitoes, as greater population reduction amplifies the impact wild-type migrants have on the long-term frequency of the anti-pathogen gene. We expect the results presented here to give pause to expectations for driving an anti-pathogen construct to fixation by relying on releasing individuals carrying this two-gene construct. Nevertheless, in some dengue-endemic environments, a spatially heterogeneous decrease in competent vectors may still facilitate decreasing disease incidence.

## Introduction

Dengue, an arbovirus vectored primarily by the mosquito *Aedes aegypti* (Linnaeus), results in approximately 390 million infections each year [1]. There is currently no vaccine available for dengue or a drug to alleviate symptoms. Consequently, vector population control remains the primary public health strategy to prevent outbreaks. Releasing genetically modified mosquitoes that carry conditionally lethal transgenes could, in principle, facilitate local vector elimination by reducing the reproductive capacity of wild females that mate with released transgenic mosquitoes. Furthermore, a recently developed female-specific lethal construct allows released individuals to transmit the transgene in the population through their male offspring (e.g., [2]–[4]). Expression of this construct is repressed by rearing mosquito larvae with tetracycline in the water (e.g., [3]), permitting the breeding of large numbers of transgenic mosquitoes for release. The transgene in the strain of [3] that only kills females when they reach the adult stage is particularly attractive because the viable larvae compete with wild-type mosquitoes during the immature stages, in addition to directly reducing the number of adults (e.g., [2], [3], [5]). This is key because density-dependent population regulation which acts during the larval stage of *Ae. aegypti* is believed to counteract population suppression efforts (e.g., as may occur in certain sterile-insect methods - [5]–[7]). Yet despite theoretical promise, analyses from detailed simulations of *Ae. aegypti* populations in realistic, urban environments predict vector population elimination may be unrealistic, and that mosquito population sizes quickly recover to previous levels if releases end [4]. Moreover, genetic control programs relying on population reduction alone are vulnerable to reinvasion if releases cease by immigrant, wild *Ae. aegypti* individuals from populations that have not been subject to control efforts [7], [8].

An alternative approach to decreasing dengue transmission is to replace wild-type populations of *Ae. aegypti* with mosquitoes carrying transgenic constructs that render them incapable of transmitting the virus (e.g., [9]–[13]). The long-term efficacy of this approach requires that the anti-pathogen transgenes spread to fixation, or at least reach and remain at a high frequency. One proposed approach to spread an anti-pathogen construct involves releasing insects carrying both anti-pathogen constructs and female-specific lethal transgenes on independent chromosomes [14]. The premise of this approach is that as the *Ae. aegypti* population size is reduced due to releasing mosquitoes carrying the female-specific lethal construct, the anti-pathogen construct will be transmitted to the population through the surviving transgenic males. Such a “reduce and replace” strategy can thus ultimately increase the frequency of the anti-pathogen construct over the time span of multiple releases. Moreover, this strategy may mitigate the risks associated with the biological or operational failure of a genetic population suppression program - even if the mosquito population returns to its pre-release density, the spread of the anti-pathogen transgene implies that most of the females would not be able to transmit the virus [14].

Field releases of transgenic mosquitoes, however, occur in spatially heterogeneous environments (e.g., [15]–[17]) with the feasibility of different release regimes depending on several factors, including regulatory issues, infrastructure, economic costs and local community support [4], [5], [18], [19]. Indeed, assessing the ultimate prospects of a “reduce and replace” strategy must consider how spatial variability in the severity of population reductions can differentially affect factors such as the extent of density-dependent interactions experienced by larval mosquitoes in different larval habitats. Here we use Skeeter Buster [20], a stochastic, spatially-explicit population model of *Ae. aegypti*, as a quantitative framework for assessing the expected efficacy of such a “reduce and replace” strategy in a realistic field environment. Using this framework, we compare the spread of an anti-pathogen transgene under different release strategies of male and female mosquitoes at different life stages. We also analyze how the presence of a fitness cost associated with the anti-pathogen construct affects prospects for population replacement. Finally, we assess how distinct spatial configurations of the releases affect the efficacy of the release strategies. Skeeter Buster has already been parameterized for the comparatively well-studied *Ae. aegypti* population in the Amazonian city of Iquitos, Peru (73.2W, 3.7S), and the model dynamics have been shown to be consistent with the major spatial and temporal patterns of pupal abundance data at the container-level from that location [21]. Thus, even though there is no planned release of transgenic mosquitoes in Peru (A. Morrison, pers. comm.), this location provides an attractive case-study for modeling the impact of a “reduce and replace” transgenic control strategy in a field setting. Although our model is parameterized specifically for the *Ae. aegypti* population in Iquitos, we believe our main conclusions could apply to other populations in tropical urban environments with limited seasonal variation.

## Materials and Methods

### Model description and parameterization

Skeeter Buster is a spatially-explicit, stage-structured model of *Ae. aegypti* that incorporates a range of processes, including temperature-dependent survival rates, container-level development and nutrient dynamics, and the impact of water level fluctuations on egg hatching (Figure S1; [20]). Skeeter Buster seeks to simulate the dynamics of an *Ae. aegypti* population across four life stages: eggs, larvae, pupae, and adults. Development of individual *Ae. aegypti* through each life stage is determined by temperature- and resource-dependent developmental rates, but is also subject to stochastic variability. Dynamics in Skeeter Buster occur on a daily time step, and individual water holding containers, along with their hydrological dynamics, are simulated for each day. Population regulation in Skeeter Buster occurs primarily during the immature stages, and adult blood meals are not considered to be a limiting resource. Water-holding containers in Skeeter Buster are assigned to specific sites on the grid, and resource dynamics within containers and the feedback between larval biomass and resource availability (i.e., density dependence) in all containers are described by the equations in [22]. Although detailed, the larval population dynamic submodels on which Skeeter Buster is based have been used in several other studies that have been calibrated to empirical data (e.g., [22]–[24]).

In Skeeter Buster, mosquitoes emerge from containers as adults and occupy sites (i.e. houses) where the containers are located. The identically sized sites are arranged on a rectangular lattice, and each site's neighbors are assumed to be described by its Moore neighborhood [25]. We do not explicitly model the spatial arrangement of containers within a site. Adults migrate during each day of their lives to an adjacent site at a fixed daily probability (default value = 0.3), with the direction of dispersal randomly determined. Skeeter Buster's dispersal submodel was calibrated to fit the mark-release-recapture experiment described in [26] (for details, see [20]). This daily movement allows a few individuals to disperse a large distance over the course of their lifetime, although this rarely occurs. To characterize rarer long range dispersal events (e.g., mosquitoes being inadvertently transported by vehicles), we also modeled long distance dispersal (over a maximum Manhattan distance of 20 sites) by a small fraction of individuals each day (default daily probability of individual movement = 0.02). In Iquitos, the distance between houses is typically between 5 and 10 meters (e.g., [21]), so this maximum long-distance daily dispersal corresponds to a mosquito moving approximately 100–200 meters in a single day. These dispersal patterns are consistent with the empirical literature (e.g., [27]). Unmated females select mates among males in the same site, and the probability of a male being selected by a given female depends on his weight. In the model, females only mate once during their lives, and in each gonotrophic cycle they oviposit their eggs into containers at the site in which they are located. Skeeter Buster is also able to model the translocation of containers between sites [20], permitting the potential migration of immature stages. However, preliminary comparisons of model runs with and without container movement under different release scenarios indicated that such movement had no qualitative effect on the outcome. Hence, for brevity, we only present detailed results for when containers were not moved between sites. Table 1 describes some of the most important parameters (as identified in [28]) as well as their numerical values. The numerical values for the entire set of parameters used in our model can be downloaded from the Skeeter Buster website (http://www.skeeterbuster.net). For a more thorough description and justification of the features, major assumptions and components of the model, see [20] and [21].

**Table 1:** Key parameters for Skeeter Buster (identified in [28]) and their numerical values.

Parameter	Description	Units	Value
A-FS	Daily baseline survival probability for adult females	day^−1^	0.89
A-MS	Daily baseline survival probability for adult males	day^−1^	0.77
A-F	Coefficient of fecundity for adult females	eggs 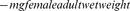	46.5
E-PTH	Temperature above which predation on eggs increases	°C	30
E-SPTH	Scalar modification to egg survival due to predation at temperatures exceeding 30°C	–	0.7
Fc†	Conversion efficiency of food to larval biomass	–	0.1
Fd1†	Coefficient governing metabolic weight loss	*mg* ^−2/3^⋅*day* ^−1^	0.016
L-S	Larval baseline daily survivorship probability	day^−1^	0.99
SD-FL	Adult female long-range dispersal probability	day^−1^	0.02
SD-FS	Adult female short-range dispersal probability	day^−1^	0.02
FC	Fitness cost of anti-pathogen transgene	copy^−1^	0.95

†. The paramters Fc and Fd1 govern larval growth dynamics and nutrient consumption within the simulated containers. Thus, in our model they contribute to the magnitude of density dependence operating within each container. For further details, see [20] and [28].

Skeeter Buster incorporates the effect of meteorological processes and the distribution and characteristics of breeding sites into its description of *Ae. aegypti* population dynamics. Thus, although most parameters in Skeeter Buster are based on laboratory or field studies from a variety of locations (e.g., [20], [28]), some parameters of Skeeter Buster must still be specified in a location-specific manner. Iquitos, Peru has been the site of intense larval habitat surveys (e.g., [29]), and such data are uniquely suited to a detailed model such as Skeeter Buster. Our analyses therefore build on prior calibration work applying Skeeter Buster to describe the population dynamics of *Ae. aegypti* at this location (e.g., [21]), and the Iquitos-specific parameters are listed in Table 2. In particular, a 2448 site area (68 sites×36 sites) is simulated with periodic boundary conditions, with the distribution of the containers in the sites parameterized according to measurements taken by the breeding site surveys following the approach taken in [21]. Assuming an average household size of 5.8 people in Iquitos (e.g., [30]), this represents a region of containing approximately 4% of Iquitos' human population. Because sites vary in the number of containers they contain, our model results in spatial variability between sites in mosquito abundances.

**Table 2:** Parameter values calibrated to Iquitos, Peru. For details, see [21].

Variable	nits	Parameter range
Daily baseline larval food input	Liver-powder equivalent per unit volume⋅day^−1^	1.32
Container-type-specific multiplier of daily food input	–	0.2–1.0
Coefficient modifying daily food input by whether the container is located indoors or outdoors	–	0.25–1.0
Daily maximal air temperature	°C	24.9–37.0
Daily minimal air temperature	°C	18.3–24.2
Daily average air temperature	°C	20.7–28.5
Daily rainfall	mm	0–271
Relative humidity index	–	100

In addition to modeling *Ae. aegypti* demographics, Skeeter Buster explicitly tracks the genotypes of all mosquitoes in the population. All loci carried by a mosquito are assumed to be diallelic and freely recombining. The allele an individual inherits from each parent at a given locus is randomly determined. Consequently, parental gametes are effectively randomly sampled, causing genetic drift to occur at lower population sizes. Because the genetic profile of the population can be explicitly followed and stochastic processes such as demographic stochasticity and genetic drift can be readily modeled, Skeeter Buster provides an attractive approach to modeling the local processes necessary to characterize the interplay between population reductions caused by the female-lethal gene and population replacement due to the spread of an anti-pathogen transgene in the declining population.

### Modeling the Release of Transgenic Strains

We model the anti-pathogen and female-specific lethal genes as two unlinked, diallelic loci. Each of the two loci is characterized by two alleles - the presence or absence of the transgene. For brevity, we refer to genotypes lacking either the anti-pathogen or female-specific lethal gene as the wild-type at that locus, while the phrase “wild-type mosquitoes” refers to individuals that lack both the conditionally lethal and anti-pathogen constructs. The anti-pathogen and female-specific lethal genes are considered to have dominant phenotypic expression. We model all released transgenic individuals as homozygous carriers of the anti-pathogen and female-killing genes. We also modeled releases of mosquitoes with four loci (two lethal loci and two anti-pathogen loci); this, however, did not substantially change the results, so we do not present the outcome of the four-locus cases.

As in [4], we assume that female carriers of the female-specific lethal genes raised in the absence of tetracycline die on the first day after they emerge from pupation. We model the transgenic strain developed by [3], whereby emerging females are unable to fly and, because they cannot locate mates or take blood meals, are effectively removed from the population upon emergence. In this strain, a transgene for a toxic protein is controlled by a two component promoter system that restricts expression to female flight muscles and completely shuts down expression of the toxin when tetracycline in the mosquito diet interacts with an engineered transcription activator protein. Thus, if female mosquitoes carrying the construct are reared as larvae in containers with tetracycline, they survive and reproduce, allowing female-specific lethality to be conditionally expressed (e.g., [31]).

Transgenes may impose fitness costs due to the expression of transgene sequences or insertional mutagenesis. For simplicity, we only analyze the case where the anti-pathogen gene carries a fitness cost, and do not model such costs associated with the female-specific lethal construct. Fitness costs could, in principle, affect any life stage, but there is no empirical data suggesting a focus on any specific stage. To retain consistency with earlier work [4], [14], [32], we assume that when present, costs associated with the anti-pathogen gene are expressed at the egg stage for both male and female mosquitoes. We assume the presence of fitness costs reduce the expected survivorship of transgenic individuals relative to wild-type individuals by 5% per copy of the transgene they carry (e.g., [4]; also this value is the midpoint of the range used in, e.g., [32], [33]). Thus, the fitness costs have the potential for removing individuals before they consume larval resources.

### Modeling the Release Regimes

Most transgenic release programs currently emphasize the exclusive release of male mosquitoes (e.g., [3]) to minimize biting nuisance and the potential risks of increased disease transmission caused by adult females. Thus, we begin by analyzing the effects of releasing only transgenic male mosquitoes. However, transgenic female mosquitoes carrying an anti-pathogen gene (with the female-specific lethal expression suppressed) cannot contribute to increased disease risk (although such releases can contribute to biting nuisance). While there are precedents for releasing female *Ae. aegypti* incapable of transmitting dengue [34], this practice is not widespread. The ability to implement this approach will depend critically on the real and perceived safety risks, such as the degree of refractoriness of the anti-pathogen transgene, and whether pathogens not targeted by the anti-pathogen gene (e.g., chikungunya) are also endemic. Hence, we also assess whether the release of transgenic females can potentially enhance both population reduction and the spread of the anti-pathogen gene compared to male-only releases.

To model the release of transgenic adults, we dynamically add cohorts of mosquitoes homozygous for both the female-specific lethal gene and the anti-pathogen gene at specified dates to specific sites. Because very large releases of mosquitoes carrying the female-specific lethal gene can cause extinctions in closed populations, particularly when population sizes are small [4], we restricted our analyses to release numbers and durations that did not always result in extinction.

*Ae. aegypti* eggs are desiccation resistant and can remain viable for long periods of time. Consequently, storing and distributing transgenic mosquitoes at the egg stage may be an attractive alternative to the distribution and release of short-lived transgenic adults that would require planning releases to coincide with individuals emerging as adults. Releases of transgenic eggs, following [3] and [4] is modeled as adding additional nutrient-filled containers into specified sites within the grid every week. Sufficient nutrient levels are provided to facilitate favorable larval development, and containers are shielded from ovipositing females. In practice, separating male and female eggs is not feasible, so for each release scenario identical numbers of male and female eggs are placed in containers. We can also compare releases of eggs that result in exclusively male adults emerging from the containers to releases of eggs resulting in both male and female adults emerging. Such bi-sex releases are modeled by releasing eggs into containers with tetracycline, suppressing expression of the female-specific lethal element among released eggs. Once all released mosquitoes die or emerge as adults, the extra containers are removed from the simulation.

In addition to varying the number, developmental stage, and sex of individuals released, we compare distinct spatial release patterns. Such comparisons can help inform the spatial configuration of releases that actual release programs should strive to reproduce. We model mosquitoes released homogenously into all sites, a regular point-source releases at every 10^th^ site along a regular grid, and random point-source releases at 10% of the sites chosen at random.

The comparison between homogenous and point-source releases also allows us to model endpoints along a continuum of mosquito dispersal behavior immediately following releases. The assumption that the dispersal behavior of recently released insects is comparable to the dispersal behavior of resident mosquitoes is standard in spatially-explicit models of transgenic control populations for vector populations (e.g., [4], [15], [35]). Nevertheless, how conditions (e.g., crowding effects from cages where males are kept immediately prior to releases) affect post-release dispersal rates remains an active area of empirical research [18], [36], [37]. We conducted a preliminary comparison between model runs that differed in the long distance adult dispersal rates immediately after releases. This comparison indicated that under elevated rates of long distance adult dispersal immediately following releases (default probability of dispersal to different site immediately following release = 0.2), the point source release regimes of male and female adults approximate homogenous release conditions of males and females. Thus, simulating a homogenous release pattern allows us to characterize the endpoint where released mosquitoes immediately disperse at elevated rates to different sites, while simulating heterogenous releases allows us to characterize the endpoint where the dispersal behavior of newly released mosquitoes does not differ substantially from that of resident mosquitoes. Alternatively, aerial releases may result in a relatively homogenous distribution of mosquitoes in all sites irrespective of potentially elevated initial dispersal behavior [4]. We further assume that distributing containers containing transgenic mosquito eggs to every site in the grid is unlikely to be practical.

In addition to the comparison between homogenous and point-source releases, we considered two different point-source configurations: a regular release pattern, where the distance between release sites is maximized, and a random release pattern, where release sites are selected at random. For instance, if sites vary in the amount of access individuals implementing the releases can expect to obtain, or if cost considerations require releases to be carried out near distribution centers located haphazardly throughout a neighborhood, the distributions of release sites may appear random with respect to the location of breeding sites. For simplicity, and to highlight the contrast between releases at a limited number of sites and releases at all sites, we assumed that the release sites would remain fixed throughout the duration of releases.

The duration of releases was also varied across the different release strategies. As noted in [4], while longer releases may facilitate a larger reduction in the population of competent vectors, they can be more difficult to sustain if community involvement and resource allocation wane [38]. Therefore, releases may be difficult to sustain indefinitely, and we followed [4] by comparing a relatively short (one year) release period to a longer (three year) release period. After releases ended, we modeled two years of population recovery to allow even the most severely reduced populations to recover towards levels approaching pre-release population sizes. Because the anti-pathogen transgene is assumed to be dominant, we assessed the efficacy of each release scenario based on the frequency of adult females carrying at least one copy of the anti-pathogen gene in the population two years after releases end. As generation times can vary between low-density and high-density contexts (for example, due to density-dependent delays in larval development) as well as due to other sources of environmental variation, we present our results in terms of absolute time rather than generation time.

Table 3 summarizes the different release strategies modeled. As Skeeter Buster is a stochastic model, we ran the model 30 times under a different spatial arrangement of sites (and hence containers) in the grid for each of the release scenarios. For the random point-source releases, we also randomized the release sites at the beginning of each simulation. Each model run was initialized with 40 wild-type eggs (20 male eggs and 20 female eggs) in all containers, and we ran the model for a single year so that the mosquito population dynamics stabilized prior to the initiation of a release program. Table S1 summarizes the initial conditions used in our simulations.

**Table 3:** The different release scenarios modeled and the corresponding figures that summarize the simulation results; when a fitness cost was associated with the anti-pathogen gene, we assumed that this reduced survivorship during the egg stage by 5% per copy of the gene (M = male-only releases, MF = releases of males and females or containers containing tetracycline, for egg releases).

Fitness cost	Life stage	Homogenous releases	Point-source (regular) releases	Point-source (random) releases	Corresponding figures summarizing results
Present	Adult	M, MF	M, MF	–	4, 5, S3
Present	Egg	–	M, MF	M, MF	6
Absent	Adult	M, MF	M, MF	–	4, 5, S3
Absent	Egg	–	M, MF	M, MF	7

**Table 4:** The release numbers and durations for the release regime described in Table 3.

Release Regime	Approximate total numbers released (1 year releases)	Approximate total numbers released (3 year releases)
Adults, all sites	2.5×10^5^–1.5×10^6^	7.5×10^5^–2.3×10^6^ (‡)
Adults, point source	1–6.6×10^6^	1–9×10^6^ (‡)
Eggs	1.2–20×10^6^ (#)	3.6–60×10^6^

(‡). In some cases, the maximum weekly release numbers for three year releases were lower than the maximum weekly release numbers for single year releases. This is because at very high weekly release numbers, conducting the releases for three years caused population extinction in all runs while in single year releases the same high weekly release numbers resulted in simulations where population extinction did not occur.

(#). Corresponds to approximately 480,000 to 8 million adults.

Finally, because strong population reductions can amplify the contribution of wild-type migrants to gene frequencies in subsequent generations, we also assessed the effect of immigration by gravid females that lack transgenes into the population. Preliminary examination (not shown) indicated the qualitative effects of immigration to be consistent across different release strategies; thus, we illustrate our results for a release strategy consisting of the release of adult males in 10% of the sites over three years, with each release site regularly spaced apart. We model immigration by dynamically adding gravid wild-type females into randomly selected sites in the urban arena.

## Results

### Releases of transgenic adult mosquitoes everywhere (homogenous releases)

**Figure 1:**
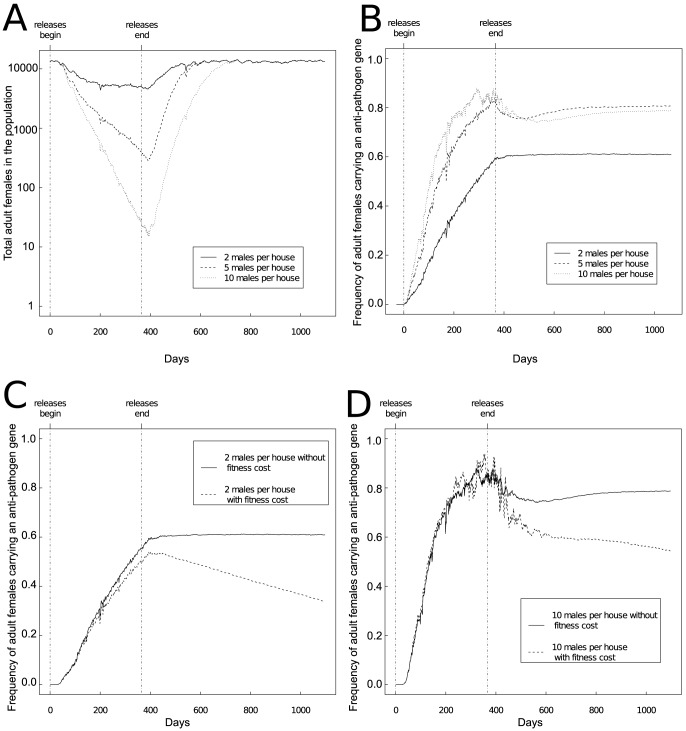
The impact of releasing an increasing number of transgenic males in all sites for a single year on (A) the population dynamics and (B–D) the frequency of adult females carrying the anti-pathogen gene in the entire simulated region. Increasing the number of males released per site per week increases the frequency of carriers of the anti-pathogen gene, but achieving fixation remains difficult. Moreover, a modest fitness cost associated with the anti-pathogen gene (5% reduction in the probability of survival per gene) substantially reduces the frequency of carriers of the anti-pathogen gene (panels C, D). The time series represent an average over 10 to 30 runs that did not result in population extinction, and dynamics from the burn-in year are not shown. The releases described correspond to total releases numbers from approximately 250,000 to 1.4 million adult mosquitoes.

We found that although releasing transgenic male mosquitoes caused considerable population reduction (e.g., sometimes much fewer than 100 adult females remaining from a population of approximately 10,000 adult females – Fig. 1A), fixation of the anti-pathogen transgene proved elusive even under homogenous releases (Fig. 1B). Comparatively modest reductions in population sizes when 2 males per site were released weekly into a total population of approximately 5000 adult males (or about 2 males per site) created conditions leading to a majority of individuals in the population carrying the anti-pathogen gene (Figs. 1A and 1B). However, even the release levels causing severe population reductions did not lead to the anti-pathogen gene being fixed in all runs (Figs. 1A and Fig. 1B). Indeed because our model is stochastic, and sites varied in the frequency of wild-type and anti-pathogen carriers, which sites had populations of wild-type individuals that went locally extinct was often randomly determined. In some simulation runs, sites with a larger number of breeding sites with wild-type individuals could, at random, not go extinct before releases end. This prevented the anti-pathogen gene from subsequently becoming fixed as sites where the wild-type individuals persisted contributed wild-type offspring during population recovery.

When relatively few males had been released and the frequency of wild-type females remained high, an anti-pathogen gene carrying a fitness cost declined slowly when releases ended (Fig. 1C). By contrast, when a comparatively large number of males were released, the frequency of an anti-pathogen gene with a fitness cost could increase at a rate comparable to an anti-pathogen gene without a fitness cost, but declined rapidly after releases ended (Fig. 1D). Moreover, the frequency of the anti-pathogen gene, particularly one with a fitness cost, was subject to strong stochastic fluctuations caused by demographic stochasticity as the mosquito population shrank and the frequency of the anti-pathogen gene increased.

**Figure 2:**
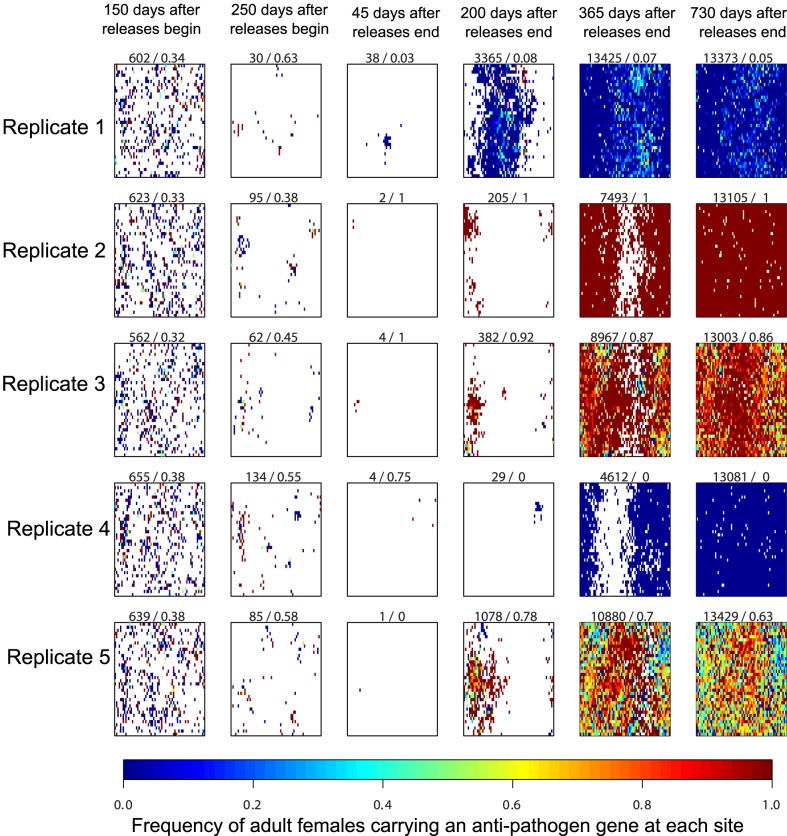
Illustrative examples of the genotypic dynamics of adult females through space and time. Here, and in Figure 3, unlike the replicate simulations in Figures 1, 4, 5, 6 and 7, we simulate the model using the same spatial configuration of potential breeding sites across replicate model runs. Results are from a simulated weekly release of 12 males in all sites for a single year (approximately 1.5 million total transgenic mosquitoes released). Colors represent the frequency of female adults carrying the anti-pathogen gene at the site, from blue (wild-type only) through red (anti-pathogen gene is at fixation). The first number at the top of each panel represents the total number of adult females in the population at the corresponding date, and the second number describes the frequency of adult females carrying the anti-pathogen gene on that date. Adult females are absent at sites without a color (white region). The prominent roles played by demographic stochasticity, spatial structure, and genetic drift are apparent in the diverse trajectories of recovery.

**Figure 3:**
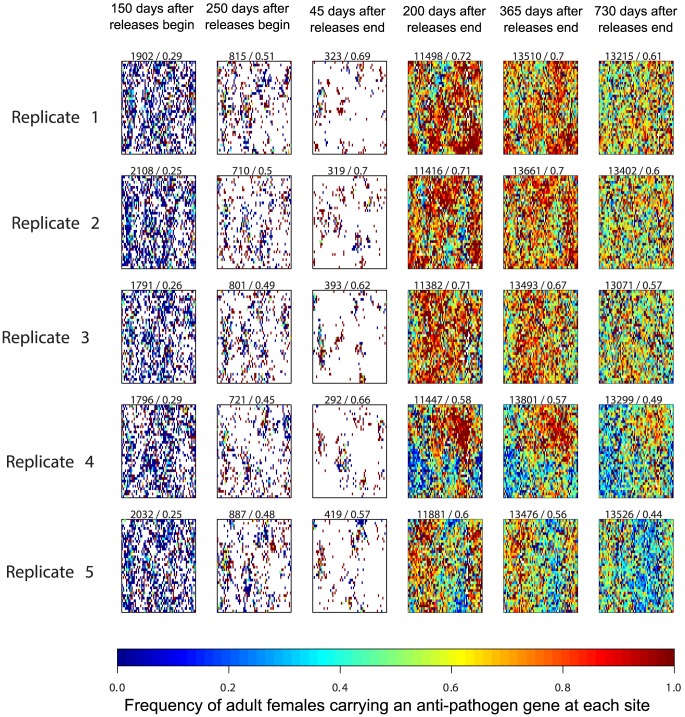
The genotypic dynamics through space given the same spatial configuration of potential breeding sites as in Figure 2, but with fewer ( = 6) males released per week (approximately 750,000 total adult transgenic mosquitoes released) and, thus, less severe population reductions. There is less variability between replicates for the overall frequency of the anti-pathogen gene two years after releases end as compared to Figure 2. As in Figure 2, the first number at the top of each panel represents the total number of adult females in the population at the corresponding date, and the second number describes the frequency of adult females carrying the anti-pathogen gene on that date. Adult females are absent at sites without a color (white region). During the recovery stage, there are spatial differences between model runs in sites that have a high frequency of carriers of the anti-pathogen gene.

Although releasing more mosquitoes increased the average frequency of female mosquitoes carrying the anti-pathogen transgene, we found that the large population reductions associated with higher release numbers also amplified the effects of genetic drift at the low population sizes that typically resulted from such releases. Figures 2 and 3 illustrate the origins of this variability; for these figures, we allowed different simulations to retain the same spatial configuration of sites, thereby isolating the effects of demographic stochasticity and genetic drift (as mentioned above, for other figures, these configurations were randomized prior to running the simulations). We found that in some model runs, the anti-pathogen gene could become fixed, but that other runs displayed a much more modest spread of the anti-pathogen gene (Fig. 2). This variability contrasted sharply with releases that did not result in such a drastic population decline (Fig. 3). Indeed, 250 days after releases begin, both Figure 2 and Figure 3 contain sites where the transgenic, anti-pathogen gene has been fixed, as well as sites where the anti-pathogen gene is entirely absent. When fewer males were released and the population reduction was not as severe at the end of releases (e.g., 45 days after releases end), both types of sites were able to contribute to population recovery (Fig. 3). This effect reduced the variability in the aggregate frequency of the anti-pathogen gene across runs. By contrast, in Figure 2 stochastic effects predominated, and whether sites where the anti-pathogen gene had reached fixation or sites where the wild-type predominated contributed to population recovery depended on which sites managed to avoid local extinction following severe population reduction. Nevertheless, even model runs with the same spatial configuration of sites and comparatively modest population reductions resulted in distinct spatial distributions of the wild-type and anti-pathogen carrying mosquitoes.

**Figure 4:**
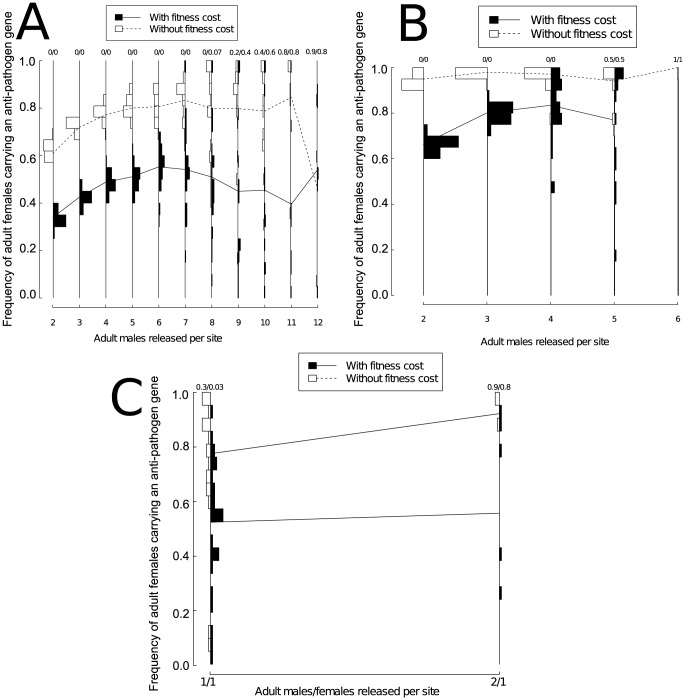
The frequency distribution across model runs for adult females carrying an anti-pathogen gene when adult male transgenic mosquitoes were released in equal numbers at all sites. Bars facing left describe results for an anti-pathogen gene that lacks a fitness cost; bars facing right describe results for an anti-pathogen gene that carries a fitness cost (5% reduction in the probability of survival per gene). For both left and right-facing bars, the frequency of adult females carrying an anti-pathogen gene increases vertically. Model runs resulting in population extinction are not shown on the histogram. Each panel describes the frequencies resulting from release programs with weekly releases at all sites for (A) a single year of male-only releases, (B) three years of male-only releases, and (C) a single year with both male and female releases. Lines between the frequency bars represent the average frequency of adult females carrying an anti-pathogen gene across runs that do not result in extinction. The first number at the top of each frequency bar represents the proportion (out of 30) of runs resulting in population extinctions without fitness costs associated with the anti-pathogen gene; the second number represents the proportion of runs (out of 30) resulting in population extinction with fitness costs associated with the anti-pathogen gene. For extinction frequencies larger than 0.1, the values have been rounded to one decimal place. Unlike the model runs illustrated in Figures 2 and 3, each model run represents a different, randomized spatial configuration of sites. Releasing one male and one female per site per week for three years always resulted in extinction. For all release strategies, increasing the number of individuals released can increase the average frequency of carriers of the anti-pathogen gene, but also increases the variability across model runs. The total release numbers range from approximately 250,000 to 1.5 million adult mosquitoes for single year releases and from approximately 750,000 to 2.3 million adult mosquitoes for three year releases (Table 4).

Higher release numbers resulted in substantially greater variability in the prevalence of the anti-pathogen gene two years after the end of releases (Fig. 4A). We found that this increased variation between model runs was true regardless of whether the anti-pathogen gene carried a fitness cost. A key consequence of this was that higher release numbers occasionally resulted in lower long-term frequencies of females carrying the anti-pathogen gene compared to simulations with lower release numbers. This occurred because the larger population reductions amplified the effects of genetic drift.

Extending the release of transgenic mosquitoes to three years facilitated the fixation of the anti-pathogen gene compared to single year releases (Fig. 4B). In the presence of a fitness cost for the anti-pathogen gene, increasing the release numbers resulted in considerable variability in the long-term frequency of females carrying the anti-pathogen gene for both release durations.

When male and female transgenic mosquitoes were released everywhere, population extinction typically occurred even at very low release numbers. This contrasted strongly with results for all-male releases (e.g., Fig. 4A). All spatially homogenous bi-sex releases for the three-year period resulted in extinction, and even for the one-year releases, releasing more than 2 males and 2 females per site always caused population extinction. The simulated populations were only able to persist when per-site release numbers were very low (less than two females or males each) and releases were restricted to a single year. Because releasing even small numbers of both males and females led to large population reductions, the long-term frequency of females carrying the anti-pathogen gene was highly variable among model runs where the populations did not go extinct (Fig. 4C).

### Point-source releases of transgenic adults

**Figure 5:**
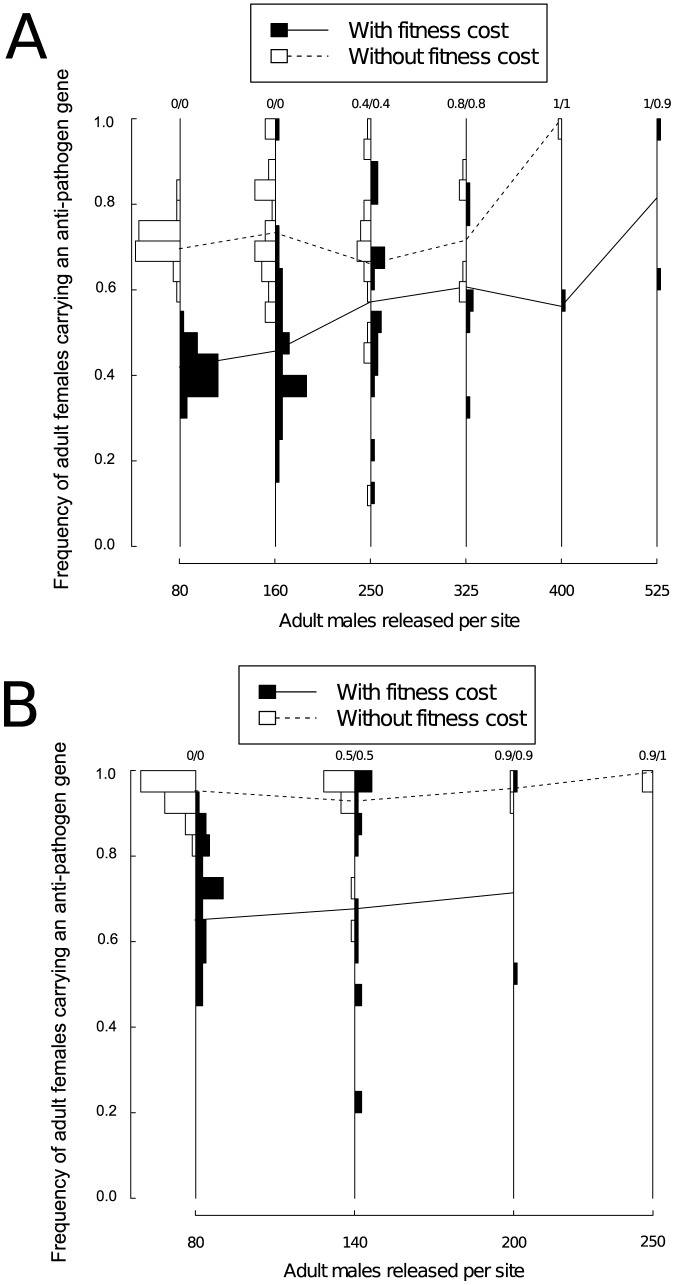
The frequency distribution across model runs for adult females carrying an anti-pathogen gene when adult transgenic mosquitoes are released according to a point-source release pattern. As in Figure 4, model runs resulting in population extinction are omitted from all panels, and the numbers at the top of each frequency bar represents the proportion of runs resulting in population extinction. The different panels represent release schedules that follow (A) a single year of male-only releases and (B) three years of male-only releases. Releasing more adults consistently raises the variability across model runs. Lines between the frequency bars represent changes in the average frequency of adult females carrying an anti-pathogen gene across runs that do not result in extinction. For extinction frequencies larger than 0.1, the values have been rounded to one decimal place. As in Figure 4, each model run represents a different, randomized spatial configuration of sites. The total release numbers range from approximately 1 to 6.6 million adult mosquitoes for single year releases and from approximately 1 to 9 million adult mosquitoes for three year releases (Table 4). Note these simulations involved higher release numbers per site compared to the case of homogenous releases (Fig. 4); this is necessary to keep the total number of released individuals comparable for both spatial release patterns.

The results above are based on an idealized release scenario where transgenic mosquitoes can be released in every site. In practice, attaining such complete coverage is challenging. As an alternative, we considered a point-source release strategy, whereby transgenic mosquitoes were released at a smaller subset (242, approximately 10%) of sites spread uniformly apart. We found that only if releases can be maintained for longer periods of time could the frequency of adult females carrying the anti-pathogen gene be raised to comparable long-term levels as when mosquitoes were released everywhere (Figs. 4A and 4B versus Figs. 5A and 5B) and even then, only if 200 or more adult males could be released weekly per site. For both one- and three-year releases, when a fitness cost was associated with the anti-pathogen gene, the long-term frequency of the anti-pathogen gene was consistently lower than when there was no such fitness cost, particularly at low release levels (Figs. 5A and 5B). For instance, for single year releases when between 80 and 160 mosquitoes were released, the frequency of adult females carrying an anti-pathogen gene at the end of two years was often lower in simulations where a fitness cost was associated with the transgene than in simulations where the fitness cost was absent. However, there was also considerable variability among runs for point-source releases. If both males and females were released (at equal densities), local extinctions were also much more likely at smaller release numbers as was the case for homogenous releases. Yet the variability between runs in the frequency of females carrying the anti-pathogen gene was large in simulations that did not result in population elimination (Figure S2).

### Point-source releases of transgenic eggs

**Figure 6:**
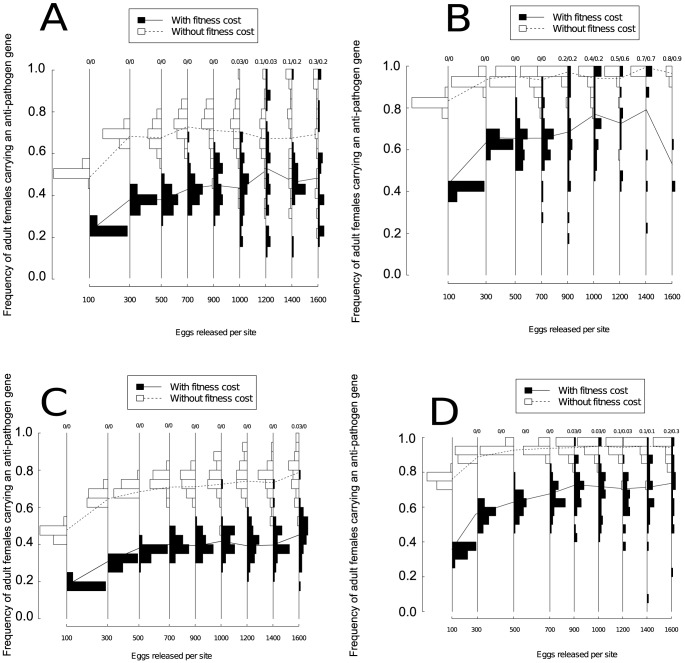
The frequency distribution across model runs for adult females carrying an anti-pathogen gene when containers containing transgenic eggs are released according to a point-source release pattern at regularly spaced sites for (A) a single year, and (B) for three years, and when the containers are released at a randomly chosen 10% subset of the sites for (C) a single year and (D) for three years. Model runs resulting in population extinction are omitted from all panels, and the numbers at the top of each frequency bar represents the proportion of runs resulting in population extinction. The spacing between release numbers on the abscissa have been adjusted to permit distinguishing the histograms. Lines between the frequency bars represent changes in the average frequency of adult females carrying an anti-pathogen gene across runs that do not result in extinction. As in Figure 4, for extinction frequencies larger than 0.1, the values have been rounded to one decimal place, and each model run represents a different, randomized spatial configuration of sites. In all panels, the total release numbers range from approximately 1.2 to 20 million mosquito eggs (Table 4).

Increasing the number of eggs released could correspondingly increase the number of runs where the frequency of the anti-pathogen gene was relatively high. However, we found that increasing the number of released eggs also substantially amplified the variability between runs (Fig. 6). This increased variability between simulations resulted in several model runs where the predicted long-term anti-pathogen gene frequencies were lower than those observed at smaller release numbers, particularly for single year releases or releases with an anti-pathogen transgene carrying a fitness cost. Thus, although the average frequency of adult females carrying an anti-pathogen gene at the end of simulations generally increased as more eggs were released, the number of simulations that resulted in a low frequency of the anti-pathogen gene also increased as more eggs were released. Eliminating the fitness cost of the anti-pathogen gene and releasing more than 1000 eggs per site each week for three years did allow the long-term anti-pathogen gene frequency to reach high levels, but in the presence of a fitness cost only a small fraction of simulation runs resulted in complete population replacement across all release scenarios. Releasing eggs at 10% of the sites determined at random at the beginning of each model simulation did not affect these qualitative results, although releasing the eggs at a random subset of the sites (Figs. 6C and 6D) resulted in fewer extinctions than releasing the eggs at regular locations. Allowing both transgenic females and males to emerge from the containers increased the fraction of model runs resulting in extinction with lower release numbers, but the qualitative results did not differ from bi-sex adult releases.

### Immigration of wild-type females

**Figure 7:**
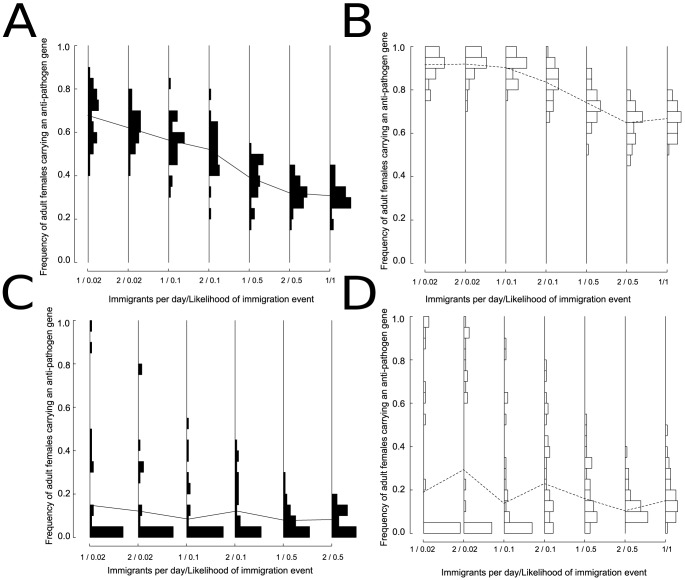
The effect of immigration on the frequency of adult females carrying an anti-pathogen gene two years after releases end under a point-source release of adult male transgenic mosquitoes with weekly releases of (A) 80 males released per site, with the anti-pathogen gene carrying a fitness cost, (B) 80 males released per site, with no fitness cost, (C) 160 males released per site, with the anti-pathogen gene carrying a fitness cost, and (D) 160 males released per site, with no fitness cost. These release numbers represent values that resulted in relatively low (80 males per site each week) and high (160 males released per site each week) levels of variability across replicate simulations in the frequencies of the anti-pathogen gene (Fig. 5A). We focused our analysis of the effects of immigration on release numbers where population extinction never occurred. These release numbers correspond to total release numbers of approximately 1 million (A–B) to 2 million (C–D) transgenic adult males over a single year. Only gravid wild-type females are assumed to migrate into the system. Although removing the fitness cost can reduce the impact of immigration, increasing the number of mosquitoes released amplifies the ability of immigration to counteract the replacement strategy. With immigration, none of the model runs result in extinction across the simulated region. As in figures 4–6, each model run represents a different, randomized spatial configuration of sites.

We found that immigration by gravid, mature wild-type females could reduce the long-term frequency of females carrying the anti-pathogen gene considerably across a range of release numbers. Increasing both the number and frequency of daily migration events lowered the average frequency of females carrying the anti-pathogen gene across runs. Moreover, the effects of wild-type migrants were most pronounced when higher numbers of transgenic mosquitoes were released per week (Figs. 7A and 7B versus Figs. 7C and 7D). This occurred because when wild-type mosquitoes immigrated into severely depleted populations, such immigrants made up a greater fraction of the population. When release numbers were smaller, if the anti-pathogen gene had an associated fitness cost, even very low rates of immigration could more than halve the average long-term frequency of transgene-carrying females compared to when a fitness cost was absent (Fig. 7A versus Fig. 7B). For larger release numbers (Figs. 7C and 7D), when immigration rates were modest (less than 2 immigrants every 10 days), there was greater variability among simulation runs, as was observed in the absence of immigration (e.g., Fig. 5A). However, even very low rates of immigration could still result in many runs where the frequency of transgene-carrying females was very low (Figs. 7C and 7D).

## Discussion

Eliminating entire *Ae. aegypti* populations by releasing engineered male mosquitoes carrying conditionally lethal elements is unlikely to be a realistic objective for large, heterogeneous urban areas [4]. Furthermore, inundative release of mosquitoes carrying an anti-pathogen transgene by itself is perceived as requiring impractically large releases (e.g., [33]). Based on results from less detailed, deterministic models [14], we anticipated that releasing transgenic mosquitoes with both an anti-pathogen gene and a conditional female-lethal gene would reduce the long term density of wild-type individuals in a population to very low levels, minimizing the risk of dengue outbreaks. Instead, exploration of a number of approaches to this “reduce and replace” strategy with a stochastic, spatially-explicit modeling framework (Skeeter Buster) indicates that in a city like Iquitos, Peru, sustaining a very low frequency of wild-type individuals could be difficult to achieve with most of the approaches we examined, particularly in the face of immigration from external populations.

Our analysis yielded insights that could not have been anticipated without explicitly considering the effects of demographic stochasticity and genetic drift at small population sizes, and how these processes affect the genetic dynamics across space and time during population recovery. Our key finding is that the strong effects of genetic drift during periods of very low population abundance and the local persistence of wild-types strongly constrain the likelihood that such a release program could drive the anti-pathogen gene to fixation (e.g., Fig. 2). These effects were not observed in our analysis of the feasibility of a “reduce and replace” strategy using a deterministic model [14]. Even modest long-term reductions in vector-competent mosquitoes are unlikely when the release areas are subject to the immigration of wild-type mosquitoes. In fact, releasing more individuals can be counter-productive, as greater population reduction amplifies the ability of wild-type migrants to determine the long-term frequency of the anti-pathogen gene.

In contrast to studies with general deterministic models, results from the stochastic, spatially-explicit model used here provide a foundation for quantifying the uncertainty surrounding different release strategies. For instance, Fig. 4A suggests that releasing more than 8 males per site for a single year can amplify the effects of genetic drift. Quantifying the uncertainty surrounding transgenic control programs is key for comparing different release strategies, particularly as the release strategies differ in their ability to cause long-term decreases in the prevalence of carriers of the anti-pathogen gene. Such reductions can be desirable when the goal of a control program is to reduce vector-competent populations below a certain threshold density to prevent the local outbreak of mosquito-borne diseases. Our results suggest that under some conditions, releasing transgenic mosquitoes that carry an anti-pathogen gene along with a conditionally lethal element can lead to long-term reductions in the abundance of vector-competent mosquitoes. For example, releasing individuals at more sites for longer periods of time (3 years instead of 1 year) and eliminating fitness costs associated with the anti-pathogen gene (e.g., Fig. 4B) could decrease the frequency of wild-type individuals.

As with all modeling approaches, however, we excluded certain considerations in the interest of tractability and space. For instance, depending on the specific anti-pathogen transgene being considered, the fitness cost may operate during different stages of the mosquito life cycle than those considered here. Our model is not meant to describe the outcome of releases involving any specific anti-dengue transgene. If experiments were to show that the fitness costs associated with a promising anti-dengue transgene operate at different stages of the life cycle, then re-evaluating our model conclusions in light of such hypothetical results would be appropriate. If such fitness costs operate primarily at later life stages (e.g., by reducing the viability of adults), then containers consisting of exclusively transgenic individuals might experience higher juvenile density dependence and potentially be less productive.

Furthermore, Skeeter Buster must be parameterized in a location-specific manner, and consequently we were only able to explore the different strategies in a specific ecological and geographical setting. Skeeter Buster has been shown to be able to characterize key spatial and temporal features of mosquito population dynamics in the location of the present study [21]. Other *Ae. aegypti* populations can exhibit considerable micro-scale heterogeneity (e.g., [39]), and thus the broad patterns we highlight, particularly with respect to the effects of genetic drift and spatial variability in constraining the ability of a “reduce and replace” strategy to cause population replacement, may still be quite informative for prospective release programs elsewhere. Nevertheless, we caution that site-specific model calibrations comparable to those carried out for Iquitos should precede attempts to identify optimal release regimes in other locations. In principle, for less intensively studied urban communities, sensitivity analyses that can account for patterns of variation between locations may be able to assess how readily our conclusions apply outside of Iquitos. For instance, the distribution of breeding containers in Iquitos may be more spatially homogeneous (e.g., [29], [40]) in comparison to other localities (e.g., Tapachula, Mexico - [41]). As noted in [4], spatial heterogeneity in local mosquito densities may inhibit population reduction. Even bi-sex releases, which we found to be quite effective at inducing population reduction under the degree of spatial heterogeneity typical for Iquitos (e.g., Fig. 4C v. Fig. 4A), may prove less effective in highly heterogeneous environments. This may reduce the efficacy of transgenic control programs based on population reduction in localities with greater between-site variability than is found in Iquitos. When locally abundant mosquito populations can serve as sources of wild-type individuals during population recovery, prospects for the success of a “reduce and replace” strategy may be diminished further.

We assumed released mosquitoes exhibit the same sex- and age-specific movement behavior as resident mosquitoes, and that for all adult mosquitoes, daily mosquito movement was mostly restricted to neighboring sites with a small fraction potentially dispersing over larger distances [20]. Our main qualitative conclusions appear robust even when individual mosquitoes are unable to disperse long distances in a single day and when daily dispersal is strictly between neighboring sites, although the transient dynamics and spatial distribution of the anti-pathogen gene differ somewhat when dispersal is more localized (Figures S3 and S4).

We found that releasing females in addition to males could allow local population extinction to occur with much smaller release numbers when females carrying the transgenic constructs were released (Fig. 4C). Indeed, if population suppression is a major goal of a transgenic vector management program, our results suggest releasing females carrying an anti-pathogen gene could greatly enhance the efficiency and efficacy of such a release program potentially without raising the risks of disease incidence. However, despite the large reduction in population sizes induced by female releases, releasing female mosquitoes in addition to males still proved insufficient to cause complete population replacement in all runs that did not result in population elimination (Fig. 4C and Figure S2). Moreover, the large population reductions female releases can cause may increase the uncertainty over the extent of population replacement and, potentially, amplify the risk that immigration could hinder efforts at population replacement. We also examined the effect of removing males from the release regimes altogether with a point-source release of female adults in every 10^th^ site. For equal numbers of females released, exclusive female releases resulted in frequencies of the anti-pathogen gene at comparable levels to when both males and females are released (Figure S2). This suggests the contribution of released males to driving the differences between male-only and bi-sex releases was quite small.

However, there are operational limitations to implementing female releases. For example, some release programs of transgenic mosquitoes heavily emphasize the exclusive release of non-biting males to assuage concerns about increased transmission risk (M. Capurro, pers. comm). Nevertheless, such female releases are not unprecedented. For instance, female mosquitoes infected with the intracellular bacterium *Wolbachia* (which can interfere with within-insect viral propagation) have been released in field settings (e.g., [34]). In the trial studies in Gordonvale, Australia, a community of approximately 670 sites where the mosquito population is seasonal, between 10,000–22,000 *Wolbachia*-infected mosquitoes were released weekly to establish *Wolbachia* infection in the population [34]. Because the spread of *Wolbachia* is expected to exhibit dynamics akin to gene-drive based transgenic control programs, fewer mosquitoes may need to be released compared to strategies that do not rely on gene-drive or similar mechanisms. Nevertheless, these release numbers are within the range of numbers we consider, suggesting that release numbers required for an effective “reduce and replace” could be higher than those suggested for *Wolbachia* releases. Hence, community acceptance of female releases at the numbers we consider may be feasible in a :“reduce and replace” context as well.

Thus, we expect the results presented here to give pause to efforts attempting to drive an anti-pathogen construct to fixation by relying on releasing individuals that also carry a conditionally female-lethal element. The amplified effect of genetic drift at small population sizes, and the spatially heterogeneous nature of population recovery in field settings, both suggest that wild-type individuals could persist in sufficient numbers to frustrate such attempts at population replacement. Although several gene-drive mechanisms have been proposed and tested in laboratory settings (e.g., [12]), only strategies based on inducing *Wolbachia* infection in all female mosquitoes have undergone field trials (e.g., [34], [42]) for *Ae. aegypti*. As transgenic control programs based on conditionally lethal constructs are unlikely to cause complete local population elimination [4], we highlight the importance of considering alternative approaches to “reduce and replace” aimed at using conditionally-lethal elements to facilitate the spread of an anti-pathogen construct. For example, releasing mosquitoes with exclusively anti-pathogen genes following a genetically-induced population reduction (“reduce-then-replace”) may permit creating conditions for an effectively high release ratio of transgenic mosquitoes carrying anti-pathogen constructs to wild-type mosquitoes for the duration of the release program. For instance, for a release regime where a single transgenic female carrying an anti-pathogen gene is released in each site each week (e.g., Fig. 4C), the weekly release ratio in the absence of population reduction preceding this release regime corresponds to approximately 0.25 transgenic mosquitoes per wild-type female when the anti-pathogen gene is first introduced. By contrast, a strategy based on population reduction alone using transgenic males could lower the adult female population size to less than 100 individuals within one year (e.g., Fig. 1). Under these conditions, switching to releases involving a single transgenic female carrying an anti-pathogen gene in each site could for the duration of the regime could result in a release ratio of approximately 25 transgenic mosquitoes per wild-type female when the replacement gene is first introduced into the population. We note, however, that the release ratio for males necessary to cause initial population reduction may still be quite high even for a “reduce-then-replace” approach (e.g., [4]). We are currently conducting a preliminary assessment of the efficacy of such a reduce-then-replace approach (Robert et al., in prep). If releasing mosquitoes carrying the conditionally lethal element can cause sufficiently large reductions, such an approach could potentially address concerns about the impracticality of inundating wild-type populations to introgress an anti-pathogen gene or the need to release large numbers of mosquitoes.

## Supporting Information

Figure S1 **A schematic depicting the main model processes described in our model (from** [**20**] **).** The biology of *Ae. aegypti* occurs within local sites, represented here with four white rectangles. In our model, eggs (E), larvae (L), pupae (P) develop within individual containers, represented by smaller white triangles, squares and hexagons square, and hexagonal polygons that could potentially describe different container types. Grey circles represent individual adult females, and orange polygons and lines characterize male adult populations and their biology. Solid lines represent development, and dashed lines represent oviposition. Dash-dotted lines represent dispersal between sites, with blue dash-dotted lines representing adult female dispersal and green dash-dotted lines representing the movement of containers between sites. Although adult dispersal and container movement can occur among all sites in the Moore neighborhood, for clarity we only depict dispersal between specific sites in this schematic. Mating occurs among adult males and females in the same site, represented by the red arrows.
(TIF)

Figure S2 **The frequency distribution across model runs for adult females carrying an anti-pathogen gene when transgenic adult mosquitoes are released according to a point-source release pattern at regularly spaced sites.** Model runs resulting in population extinction are omitted from all panels, and the number at the top of each frequency bar represents the proportion of runs resulting in population extinction. The different panels represent releases of adults for (A) three years with only female adult releases, (B) a single year of only female adult releases, (C) three years of male and female releases, and (D) a single year of male and female releases. The frequency distribution for the female-only and male and female releases are quite similar, suggesting a limited impact of released males when females are released. Lines between the frequency bars represent changes in the average frequency of adult females carrying an anti-pathogen gene across runs that do not result in extinction. As in Figure 4 in the main text, for extinction frequencies larger than 0.1, the values have been rounded to one decimal place, and each model run represents a different, randomized spatial configuration of sites. The release numbers correspond to approximately 190,000 to 1.9 million total transgenic mosquitoes released.
(EPS)

Figure S3 **Illustrative examples of the genotypic dynamics of adult females through space and time given the same spatial configuration of potential breeding sites across replicate model runs.** Results are from a simulated weekly release of 12 males in all sites for a single year in the absence of daily long range dispersal and under absorbing boundary conditions (approximately 1.5 million total transgenic mosquitoes released). Colors represent the frequency of female adults carrying the anti-pathogen gene at the site, from blue (wild-type only) through red (anti-pathogen gene is at fixation). The first number at the top of each panel represents the total number of adult females in the population at the corresponding date, and the second number describes the frequency of adult females carrying the anti-pathogen gene on that date. Adult females are absent at sites without a color (white region). The prominent roles played by demographic stochasticity and genetic drift are apparent in the diverse trajectories of recovery, including complete population replacement in some runs. We note that unlike the corresponding figures in the main text, sites along the boundary receive transgenic individuals from only three of their four neighboring sites (due to the absorbing boundary condition assumption), allowing persistence of these sites. Moreover, daily mosquito movement was restricted only to neighboring sites in these runs, resulting in greater clustering of the genotypic frequencies in space. Thus, model runs with very severe bottlenecks (e.g., Replicate 2, panels E–H), complete population recovery was not as rapid as model runs where the bottlenecks were not as strong (e.g., Replicate 5; panels Q–T).
(EPS)

Figure S4 **The genotypic dynamics through space given the same spatial configuration of potential breeding sites as in Figure S3, but with fewer ( = 6) males released per week (approximately 750,000 total adult transgenic mosquitoes released) and, thus, less severe population reductions.** The spatial trajectories of recovery differ considerably between runs, reflecting the influence of demographic stochasticity, spatial heterogeneity, and genetic drift; however, there is less variability between replicates for the overall frequency of the anti-pathogen allele. Nevertheless, even two years after releases end, considerable spatial differences persist between model runs in sites that have a high frequency of carriers of the anti-pathogen allele as a consequence of limited long-range dispersal and the use of non-periodic boundary conditions.
(EPS)

Table S1 **A summary of initial conditions for the model.**
(PDF)
