# Supplementary figures and images for: A Reduce and Replace Strategy for Suppressing Vector-Borne Diseases: Insights from a Stochastic, Spatial Model
Source: PLoS One. 2013 Dec 20;8(12):e81860. doi: 10.1371/journal.pone.0081860 (PMC3869666; doi:10.1371/journal.pone.0081860)

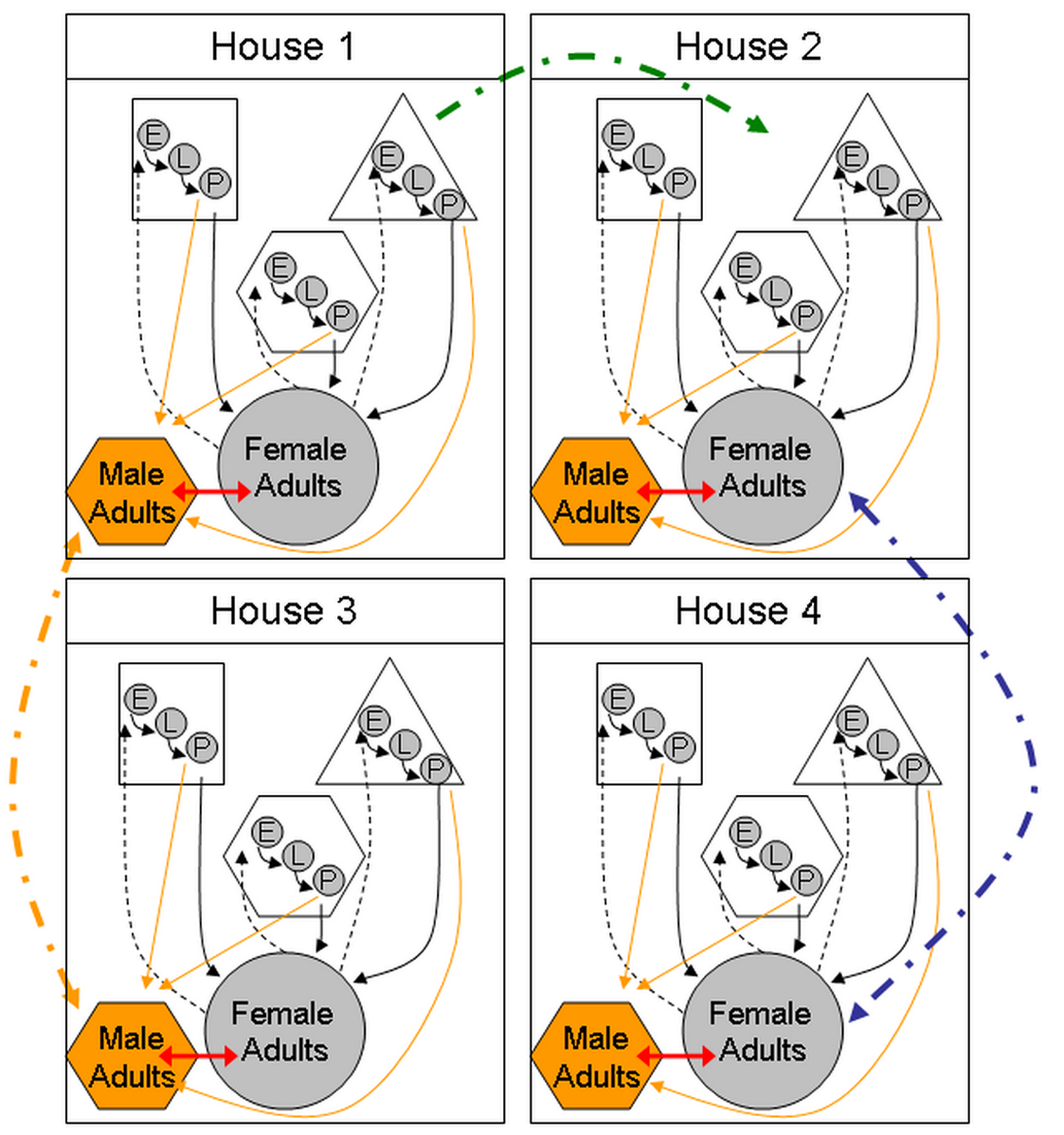

Supplement: Figure S1 — A schematic depicting the main model processes described in our model (from [20] ). The biology of Ae. aegypti occurs within local sites, represented here with four white rectangles. In our model, eggs (E), larvae (L), pupae (P) develop within individual containers, represented by smaller white triangles, squares and hexagons square, and hexagonal polygons that could potentially describe different container types. Grey circles represent individual adult females, and orange polygons and lines characterize male adult populations and their biology. Solid lines represent development, and dashed lines represent oviposition. Dash-dotted lines represent dispersal between sites, with blue dash-dotted lines representing adult female dispersal and green dash-dotted lines representing the movement of containers between sites. Although adult dispersal and container movement can occur among all sites in the Moore neighborhood, for clarity we only depict dispersal between specific sites in this schematic. Mating occurs among adult males and females in the same site, represented by the red arrows. (TIF) [file pone.0081860.s001.tif]
