# Supplementary material for: A Reduce and Replace Strategy for Suppressing Vector-Borne Diseases: Insights from a Stochastic, Spatial Model
Source: PLoS One. 2013 Dec 20;8(12):e81860. doi: 10.1371/journal.pone.0081860 (PMC3869666; doi:10.1371/journal.pone.0081860)
Supplement: Table S1 — A summary of initial conditions for the model. (PDF) [file pone.0081860.s005.pdf]

Supplementary Table S1. Summary of initial conditions for the model.

Variable	Value
Number of sites	2448
Percent mosquito infested containers	100
Number of eggs per container	40
Number of larvae per container	0
Number of pupae per container	0
Number of adult mosquitoes per house	0
Initial sex ratio	1:1
Boundary conditions	Periodic
Wild-type frequency	100
